# Mammary stem cells: angels or demons in mammary gland?

**DOI:** 10.1038/sigtrans.2016.38

**Published:** 2017-01-13

**Authors:** Xueman Chen, Qiang Liu, Erwei Song

**Affiliations:** 1 Breast Tumor Center, Sun Yat-Sen Memorial Hospital, Sun Yat-Sen University, Guangzhou, China; 2 Guangdong Provincial Key Laboratory of Malignant Tumour Epigenetics and Gene Regulation, Medical Research Center, Sun Yat-Sen Memorial Hospital, Sun Yat-Sen University, Guangzhou, China

## Abstract

A highly dynamic development process exits within the epithelia of mammary gland, featuring morphogenetic variation during puberty, pregnancy, lactation, and regression. The identification of mammary stem cells (MaSCs) via lineage-tracing studies has substantiated a hierarchical organization of the mammary epithelia. A single MaSC is capable of reconstituting the entirely functional mammary gland upon orthotopic transplantation. Although different mammary cell subpopulations can be candidate cells-of-origin for distinct breast tumor subtypes, it still lacks experimental proofs whether MaSCs, the most primitive cells, are the ‘seeds’ of malignant transformation during most, if not all, tumorigenesis in the breast. Here, we review current knowledge of mammary epithelial hierarchy, highlighting the roles of mammary stem/progenitor cells and breast cancer stem cells (BCSCs) along with their key molecular regulators in organ development and cancer evolution. Clarifying these issues will pave the way for developing novel interventions toward stem/progenitor cells in either prevention or treatment of breast cancer (BrCa).

## Introduction

Human breast cancer (BrCa) is a highly heterogeneous disease. In terms of gene expression profile, ~18 histological and at least five molecular subtypes can be characterized to classify breast tumors.^1–3^ Given that tumorigenesis is, in essence, a deregulated organogenetic disorder, there might be normal mammary epithelial counterparts that parallel to cancer cells.^4^ Accumulating evidence has shown that different tumor subclasses might derive from distinct cell subpopulations within the mammary epithelia.^5–8^ As such, elucidating normal epithelial differentiation hierarchy is helpful to understand BrCa heterogeneity and to identify the potential cancer cells of origin.

Mammary gland development in humans and mice takes place predominantly after birth. Structurally, mammary glands are constructed of ducts and lobules lined by hierarchical cells that range from stem cells to progenitors to terminally differentiated cells.^4^ Mammary stem cells (MaSCs), located at the top of epithelium hierarchy, possess hallmark properties including self-renewal and multi-directional differentiation. Transplantation assays along with limiting dilution assay (LDA) have demonstrated that one individual MaSC can recapitulate a complete mammary gland that exhibits full developmental capacity *in vivo.*^9,10^ Progenitor cells comprising at least three subtypes are characterized by their proliferative potential. In the context of cancer, albeit still under controversy, both stem cells and progenitor cells are candidate cells-of-origin in tumorigenesis.

In this review, we will provide an overview of mammary gland development in both humans and mice, highlighting the differentiation hierarchy where mammary stem/progenitor cells may serve as cellular origin in BrCa. In addition, we will present a brief introduction of breast cancer stem cells (BCSCs), as well as critical molecular regulators involved.

## Mammary gland ontogeny: stem cells on the right track

**Figure 1:**
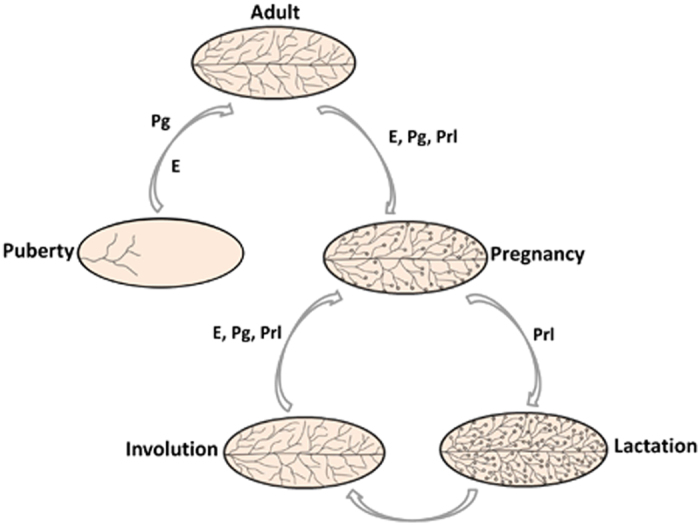
Mouse mammary gland develops postnatally under the control of systemic hormones. See text for detailed descriptions. E, estrogen; Pg, progesterone; Prl, prolactin.

The mammary gland is a unique organ featuring postnatal development in that most of its patterning will not occur until adulthood, both in mice and in humans.^11^ A female mouse has five pairs of mammary epithelial placodes since embryogenesis. The rudimentary placodes derive from the ectoderm at embryo stage, gradually penetrate to shape mammary buds which sprout with a lumen.^12^ A week later, a small underlying mammary branch begins to invade the developing fat pad but keeps in a restricted pace even after birth within three weeks. As the level of estrogen arises during puberty, gland expands and comes with profound morphogenesis—a branching, bilayer ductal structure formed by an outer basal layer of myoepithelial cells, whose contraction allows milk secretion, surrounding an inner luminal layer of cells, which comprise ductal luminal cells (LCs) lining the inside of the ducts and alveolar LCs to produce milk at parturition.^13^ The pubescent mammary gland growth is mainly driven by specialized club-like structures termed terminal end buds (TEBs), which localize at the distal tip of the ducts and lead invasion through the empty adipose tissue until reaching the end of the mammary fat pad (MFPs).^14^ TEBs are subdivided into an outer layer of ‘cap’ cells (functionally referred to as MaSCs) and an inner layer of ‘body’ cells, whose progeny are traced to be myoepithelial and luminal cells, respectively.^15^ The side branching of young adult mammary gland is controlled by progesterone, while during pregnancy, systemic hormones including estrogen, progesterone and prolactin work in concert to induce alveolar expansion, resulting in structural remodeling of the gland.^16^ Collectively, highly ramified ducts proliferate and differentiate into secretory lobuloalveolar buds with each estrous cycle. In the late pregnancy and during lactation, mammary epithelia almost fill the MFP and prolactin functions to establish the secretory state. Alveolar cells secrete milk into the lumens under the contractile force of myoepithelial cells along with the ducts. At the end of lactation, the lobuloalveoli undergo regression and the gland returns to a virginal appearance.^17^ When it comes to another round of pregnancy, the well-choreographed processes comprising proliferation, differentiation and involution re-emerge, thereby constituting a successive reproductive ‘mammary cycle’ (Figure 1).^18^

Analogous to TEBs in mouse mammary gland, the main lobular structure of human breast is named terminal ductal lobular units (TDLUs) where the branching ducts terminate and most breast tumors arise.^19^ Throughout development and pregnancy, TDLUs exist in dynamically diverse morphological forms, varying from undifferentiated Lob1-type state (in the virgin gland) to differentiated Lob2 and Lob3 with more ductules, further Lob4 secretory acinar structures (during pregnancy), ultimately regressing back in Lob2 type (after parturition). It is worth noting that Lob1 structures are the most predominant population (with a moderate level of Lob2 rather than Lob3/Lob4) in nulliparous or post-menopause women.^20,21^ However, the human breast covers more fibrous connective tissue while the mouse mammary tissue owns a larger number of adipocytes,^19^ thus making it different in transplantation assays.

Despite morphogenetic differences in the ductal tree, there are striking functional parallels between mouse and human mammary tissue, as supported by breast tumorigenesis in genetically engineered mouse models.^19^ The consistent massive expansion of mammary epithelia that occurs during puberty and pregnancy as well as each reproductive cycle further points to a stem-like cell, namely MaSC, with inherent longevity and remarkable regenerative capability residing in the mammary epithelia.^22^ Emerging evidence has shown that MaSCs serve as a pioneering but good force in both mouse and human mammary gland development and organ homeostasis maintenance, until they go awry in cancer.

## Identification of MaSCs

To begin with the definition of MaSC, it is traditionally characterized as a cell that can self-renew to maintain the stem cell pool, despite massive cell apoptosis post weaning, and can differentiate into mature epithelial cells of either myoepithelial or luminal lineage via a series of lineage-restricted progenitor intermediates. Both symmetric and asymmetric divisions contribute to MaSC self-renewal while the latter generates more differentiated progeny—morphologically distinct progenitor cells with proliferative potential towards two main terminally differentiated cells that construct the entire mammary epithelia. Apart from self-renewal and directional differentiation, long-term survival and expansion of MaSCs may allow increased susceptibility to neoplastic transformation.^23^ In addition, sequential accumulation of deleterious genetic and/or epigenetic alterations in MaSCs that persist over their whole lifespan, may render them as vulnerable targets of BrCa formation or relapse.^24^ Thus the tumorigenicity of MaSCs needs further consideration and more in-depth investigation, though many technical obstacles lie in reality.

**Figure 2:**
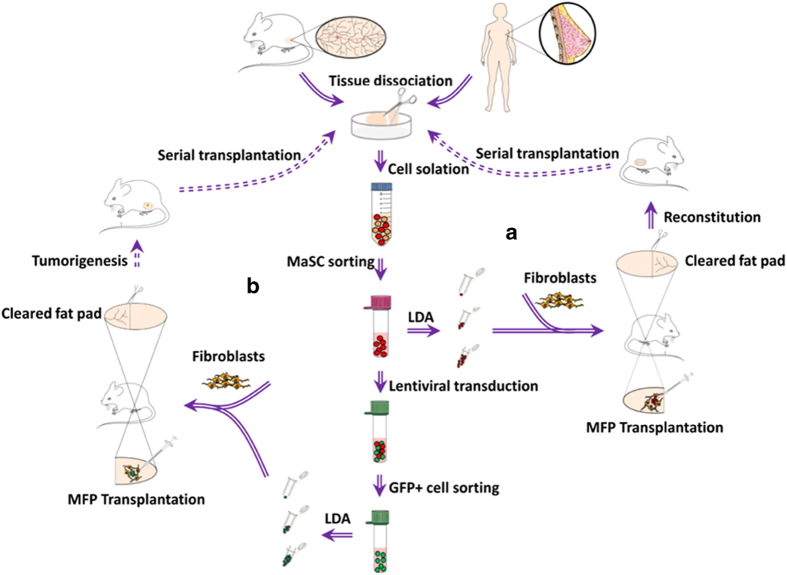
Schematic for mammary fat pad transplantation. (**a**) mammary gland reconstitution. (**b**) MaSCs upon oncogenic transformation as cell-of-origin model (albeit unconfirmed). In both cases, fibroblasts are co-injected with human MECs for humanization.

The discovery of MaSCs dates back to the late 1950s, when the serial transplantation assay was originally applied in de-epithelialized MFPs of syngeneic mice for mammary gland reconstitution (see Figure 2a for schematic of MFP transplantation). To date, it’s still the gold standard for stem cell assays. The pioneering work from DeOme *et al.* showed that portions of the normal mammary epithelia from donor mice, when transplanted into recipient fat pads cleared of endogenous epithelium, could reproduce an entire functional mammary epithelial tree.^25^ The epithelium-free MFPs of mice allowed *in-situ* transplantation and growth of normal, pre-neoplastic and malignant mammary tissues, leading to repopulation of normal mammary gland and development of mammary tumors, respectively. Successful engraftments obtained from randomly distributed cells within the mammary gland at any developmental stages further implied the existence of widespread repopulating cells.^26–28^ Subsequent studies have demonstrated that the reconstitution ability in the mammary gland was ascribed to the proliferative activity of a single cell with stem-like phenotypes, inferred to be MaSC. Moreover, the progeny of primary transplanted cells exhibited serial transplantability at a clonal level to generate ductal-lobular epithelial outgrowths. Unlike pre-neoplastic/neoplastic cells to be almost unlimitedly passaged, the normal ones always undergo senescence after finite (generally five to eight) transplant generations.^27,29^ Operationally, cells with these properties were termed mammary repopulating units (MRUs) or simply MaSCs, the former of which is actually more preferable except that the outgrowth is definitively progeny of a transplanted single microscopically visualized mammary epithelial cell (MEC).^30^

As stem cells exist in the mammary gland, plenty of strategies are thereafter developed to identify and purify MaSCs based on their morphological or biological properties.^13^ Previously, the ‘cap’ cells that line the outside of the TEBs, and the pale or light-staining cells with low cellular complexity (that is, few cytoplasmic organelles),^26^ which were afterwards known as undifferentiated large light cell (ULLC) and small light cells (SLCs) by electron microscopy, were hypothesized to represent the undifferentiated mammary stem/progenitor cell population. Later on, label retention experiments identified mouse MECs that retained their template DNA strands during asymmetric division harbored stem cell characteristics.^31^ However, no direct evidence for regenerative capacity had ever been presented for cells isolated via these approaches. Also, the side-population cells defined by Hoechst 33342 dye efflux, though once stand for the MaSC-enriched fraction,^32,33^ has been found abundant in luminal progenitor population.^22^

## Prospective isolation of MaSCs

To better enrich for cells with stem cell characteristics, fluorescence-activated cell sorting (FACS) is employed, and according to the expression of specific cell-surface makers, all kinds of cell subsets including MaSCs can be isolated from freshly dissociated mammary gland preparations. Stem cell antigen-1 positive (Sca1^+^) cells used to be identified as a subpopulation of label-retaining MECs and to some degree capable of mammary reconstitution. In 2006, it was reported in *NATURE* that mouse MaSCs could be recognized and highly purified by cooperated makers—CD24 (heat-stable antigen) and CD29 (β1-integrin) or CD49f (α6-integrin).^9,10^ It was the first time to provide functional evidence that a single cell within the Lin^−^CD24^+/med^CD29^hi/^CD49f^hi^ but exceptionally Sca1^low/−^ population, when orthotopically transplanted into mice at limiting dilutions, displayed the stem cell hallmark features of self-renewal and multi-lineage differentiation. Subsequent studies confirmed the above-mentioned molecular phenotype for effective enrichment of putative MaSCs.^34,35^ Another breakthrough came in 2015 when protein C receptor (Procr), a novel Wnt target in mammary epithelia, was found able to mark a rare unique subset of multipotent mouse MaSCs via lineage tracing.^36^ Lineage-tracing assay is a method most commonly used in combination with genetically engineered mouse models to address putative cell-of-origin of various tumors, whose outcome is determined by the ‘all-or-none law’—all tumor cells/an entire tumor or not, by labeling and tracking target cells and their progeny *in vivo*. Procr-expressing basal cells out-competed total CD24^+^CD29^hi^ basal cells in increased *in vitro* colony-forming efficiency and in extraordinary *in vivo* repopulating activity upon implantation, representing a highly purer population of MaSCs. The emerging surface maker profile of mouse MaSCs thus far is Lin^−^Procr^+^CD24^+/med^CD29^hi^CD49f^hi^Sca1^low/−^.

In regard to the detection of human MaSCs, they were poorly purified compared to mouse counterparts due to a lack of reliable makers. Initially, the technology ‘mammosphere culture’ was developed in nonadherent conditions, following the example of neural stem cell-enriched ‘neurospheres’.^37^ Unfortunately, the yield of MaSCs is less than 1% from such cultured mammospheres. To further refine this approach, a lipophilic fluorescent dye PKH26 was used to label cells with slow-cycling and quiescent traits during mammosphere growth.^38^ Subsequent FACS for those retaining this label, followed by *in vivo assay* in humanized mouse mammary glands, further confirmed their stem cell nature.^38^ Cell-surface markers also hold promise for the purification of human MaSCs. Studies showed that Lin^−^CD49f^+^EpCAM^neg–low^ or CD10^+^ basal phenotype could enrich for human MRUs, which exhibited reconstruction ability when transplanted into subrenal capsule or cleared MFPs of NOD-SCID mice that had undergone fibroblast-associated ‘humanization’, a supplementary procedure to reestablish a stromal environment characteristic of that in human breast tissue (Figure 2a).^39,40^ Nevertheless, distinct strategies often accompany with diverse results. For example, one report indicated that cells with repopulating potential in humanized mouse MFPs were derived only from the aldehyde dehydrogenase 1-positive (ALDH1^+^) cells.^41^ This is in contrast with another study that outgrowths beneath renal capsule were restricted to the ALDH1^low^ basal cell compartment.^42^ In addition, it remains controversial whether human mammary stem/progenitor cells reside in only basal epithelial subset^39,40^ or both luminal and basal cell populations.^43^

## Moleculat regulators of MaSC signaling pathway

**Figure 3:**
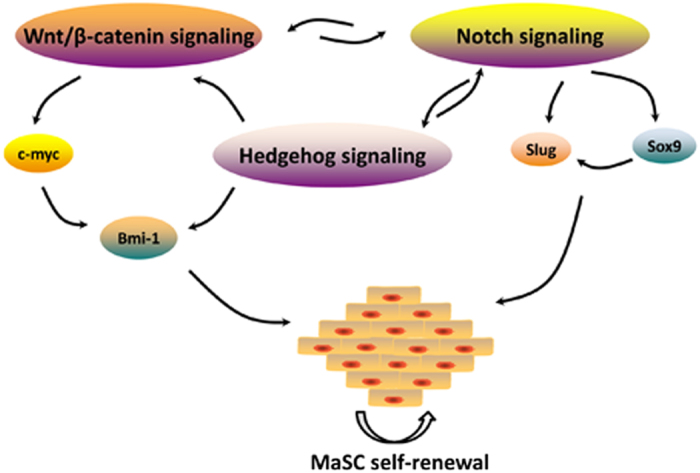
A simplified signaling pathway network where Wnt/β-catenin, Notch and Hedgehog pathways, along with critical transcription factors including Bmi-1, c-myc, Slug and Sox9, interact with one another and contribute to the maintenance of MaSC state.

Based on the purification approaches for MaSCs, subsequent experiments were undertaken to unravel the molecular mechanisms that govern MaSC ‘stemness’ and differentiation along a particular lineage. Both Wnt/β-catenin and Notch are classical signaling pathways in regulating MaSC fates. The Wnt receptor LRP5 is the first single biomarker to some extent to enrich for MaSCs, and more importantly, functionally involved in stem cell maintenance.^44^ A 6.4-fold increase was observed in the absolute number of MaSCs from the MMTV-wnt-1 transgenic mice,^9^ and Wnt3A-treated MaSCs exhibited a competitive advantage to repopulate the mammary gland,^36,45^ conferring Wnt proteins as self-renewal factors for MaSCs. Notch signaling plays an active role in different developmental stages of mammary gland, generally starting with the asymmetric cell fate determination.^46^ It has been shown that MaSCs are Notch signal-generators with the ligands expressed on their surface, while the downstream progenitor/luminal subtypes expressing Notch receptors receive the signals.^38,47^ Endogenous Notch signaling restricts the renewal of MaSCs,^47^ and the tumor suppressor p53 arrests MaSC expansion as Notch does.^48,49^ ∆N-p63, an isoform of the basal-restricted p63 transcriptional factor, exerts opposite effect. Specifically, its expression in MaSCs induced by Wnt signaling^44^ contributes to stemness maintenance, whereas its downregulation via Notch proteins^50^ is predisposed to luminal lineage commitment.^51^ Furthermore, Slug and Sox9 work in concert to determine MaSC state.^52^ Other potential molecular pathways or transcriptional modulators involve Hedgehog, Bim-1, c-myc and so on, all of which affect MaSC activity either *in vitro* or *in vivo.*^53,54^ Collectively, a complex signaling pathway network underlies the self-renewal and lineage commitment of MaSCs. Figure 3 delineates that the Hedgehog, Notch and Wnt/β-catenin signaling pathways form a loop where Notch and Hedgehog or Wnt pathways feature bidirectional regulation. Notch signaling governs Slug and Sox9 in a closed-loop, and Bmi-1 serves downstream of Wnt-mediated c-myc or Hedgehog signaling directly, all of which contribute to MaSC self-renewal. However, the signals or effectors conducting normal mammary development are frequently subverted in cancers.

## Differentiation hierarchy and implications for breast tumorigenesis

As mentioned above, a differentiation hierarchy within the mammary epithelia is constructed as mammary gland develops. A stem cell can asymmetrically segregate into an identical progeny and a committed progenitor cell. Multi-lineage differentiation of mammary epithelial progenitors also exists in normal adult human breast. Bipotent progenitors are supposed to yield myoepithelial and luminal progenitors. On the one hand, the myoepithelial progenitor subpopulation differentiate into highly elongated myoepithelial cells that reside in a basal position; on the other hand, the luminal progenitor cells (LPCs) commit to either ductal or alveolar sublineage at distinct developmental stages—puberty or pregnancy, respectively.^19^

Understanding the normal cellular hierarchy in mammary epithelia is an important prerequisite to identify the cells-of-origin in BrCa. There are at least five definitive molecular subtypes in BrCa, including luminal A, luminal B, HER2-positive, basal-like, and ‘claudin-low’ or ‘normal-like’.^19,55,56^ Based on the gene expression patterns, all these subclasses are largely determined by the presence or absence of ER or PgR, and the amplification/overexpression of HER2/ERBB2 locus.^1,2,55^ The idea existed for long that transformed basal stem/progenitor cells gave rise to basal-like BrCa with high levels of basal cell markers such as K5 and K14, while luminal subtype expressing high levels of LC markers (e.g., K8 and K18) arose from LPCs.^57^ However, gene expression profiling of different mammary cell subpopulations uncovered similarities to specific subtypes of BrCa, revealing a new perspective of relationship between human breast epithelial hierarchy and cancer subclasses.^5,6^ In this comparative molecular study, the MaSC/basal cells featured a gene signature closest to the ‘claudin-low’ and ‘normal-like’ rather than basal subtype, which was reversely most concordant with the luminal progenitor signature. In addition, the relatively mature LC signature genes shared more similarities to luminal A and B profiles. However, due to the existence of dedifferentiation state or cell plasticity during neoplastic development, a small progenitor subset within mature populations is not exclusively the real cellular target.^16^ And more experimental clues are needed to address cell-of-origin for the HER2-positive subtype.

Notably, LPCs have been the best-known candidate of cellular origin for BRCA1-associated basal-like BrCa. LPCs can be distinguished from MaSCs via a combination of cell-surface markers, displaying a CD29^lo^CD24^+^CD61^+^ phenotype in mice or EpCAM^hi^CD49f^+^ in humans. Various studies have revealed their colony-forming ability *in vitro*. As mentioned, pre-neoplastic human tissue from BRCA1 mutation carriers harbored an expanded luminal progenitor population whose expression profiles are closely aligned with that of basal tumors arising in BRCA1 heterozygous women.^5^ Subsequent studies concerning genetic predisposition of progenitor cell transformation and BRCA1/p53-deficient transgenic mouse models further indicate a luminal-to-basal mammary tumor conversion under BRCA1-mutated background.^7,58,59^

**Figure 4:**
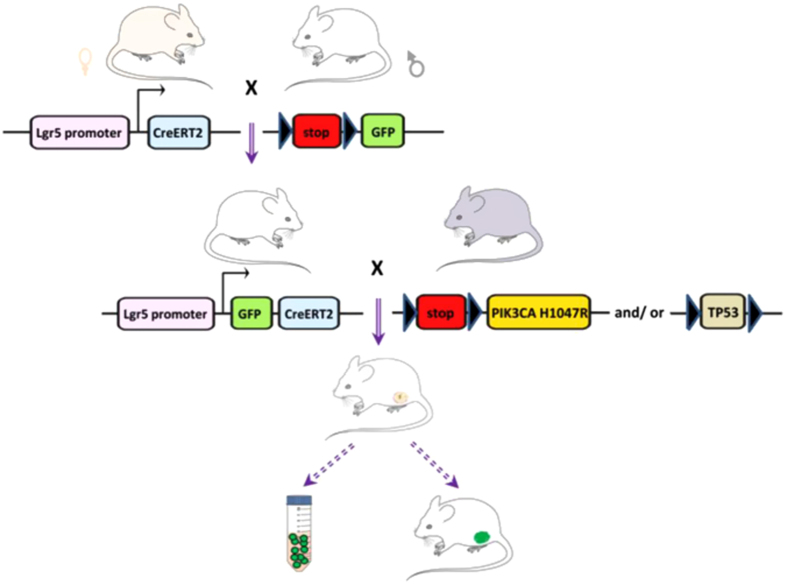
Schematic diagram of Cre-loxP system-based genetically modified mice using lineage-tracing assay to track cellular origin of cancer. The double dotted lines arrow unproved results. PIK3CA-targeted and/or TP53-deleted MaSCs with GFP labeling are supposed to generate a totally GFP+ mammary tumor with all the cells labeled by GFP.

In concert with cells of origin, initiating genetic alterations contribute largely to the molecular profile of BrCa, for example, depletion of BRCA1/2 in any of the tested cell populations initiates basal-like while PTEN knockout causes normal-like cancers.^60^ The oncogenic PIK3CA^H1047R^ mutant expression, along with TP53 deletion or not, in lineage-committed basal (Lgr5, K5 or K14-Cre mouse model; see Figure 4 for detail schematic representation) or luminal cells (K8-Cre mouse model) triggers dedifferentiation of cells into a multipotent stem-like state, generating luminal-like or basal-like cells, respectively, thereby leading to the development of multi-lineage mammary tumors with intratumoural heterogeneity.^61,62^

Regarding MaSCs-of-origin, their correlation with tumorigenesis is thus far supported by the fact that their absolute counts increased in premalignant mammary tissue from MMTV-wnt-1 mice.^9^ Although infrequently reported, they can’t be simply excluded. As scientists recently demonstrated that only oncogene-targeted stem cells, but not progenitor cells, were responsible for the induction of basal cell carcinoma upon hedgehog activation,^63^ it might not take long to unveil whether MaSCs behave during tumor initiation. In fact, there are some technological problems in directing oncogenic lesion into MaSCs due, in part, to their rarity, slow-cycling state and absent specific markers. Figure 2b proposes a schematic to address the tumorigenic capacity of MaSCs upon lentivirus-delivered oncogenic transformation followed by cleared MFP transplantation. Theoretically, any mammary cell population with proliferative potential can be candidate targets for transformation, only if it obtains mutations that revoke regenerative capacity and block the access to differentiate into a post-mitotic state.^64^

## Breast cancer stem cells (BCSCs) as devil leaders in cancer onset

Cancer stem cells (CSCs) are mainly characterized by their potential of self-renewal and multipotency, which are typically accepted as stem cell hallmarks.^65^ As reflected by their alternative terms such as tumor-initiating cells (TILs) or cancer-propagating cells, CSCs are responsible for most, if not all of the onset of tumorigenesis, as well as the maintenance of tumor propagation.^64,66,67^ Human breast tumors harbor a small cell fraction named BCSCs with features reminiscent of normal MaSCs, and uniquely, holding tumorigenic property. The identification of CSCs in BrCa can be traced back to 2003 when a CD44^+^CD24^−/lo^^w^ population was found able to generate heterogeneous tumors upon serial transplantation into immune-deficient hosts.^68^ The BrCa cell hierarchy of which CSCs located at the apex remains unclear relative to the normal ones.

It was long believed that BCSCs are derived from normal stem cells, which acquire heritable changes like somatic mutation, and function as intermediate between transformed MaSCs and cancerous breast. However, contrasting evidence has emerged that normal MaSCs arise from the basal layer of mammary epithelia while breast TILs reside in the luminal layer, both of which are under the control of distinct epithelial-to-mesenchymal transition (EMT) programs.^69^ Given that CSCs are not directly arisen from normal stem cells, scientists have put up with a plastic model of tumorigenicity that transit-amplifying cells, often termed progenitor cells, can initially acquire somatic alteration or heritable epigenetic changes, and then pass them onto CSC population by self-dedifferentiation.^70^ In this way, progenitor cells may be the actual targets of oncogenic events, followed by plasticity-induced progenitor dedifferentiation that can give rise to CSCs, resulting in tumor initiation or metastatic dissemination. Thus, progenitors rather than stem cells within a tumor should be more preferable therapeutic targets in clinical practice.

## Noncoding RNAs regulating stemness and differentiation

Mounting studies of long and short noncoding RNAs (ncRNAs) help unveil mechanisms of the stemness maintenance of both MaSCs and BCSCs, the organogenesis in mammary gland, as well as the BrCa onset and development.^71^

MicroRNAs (miRNAs) are well-known as posttranscriptional negative gene regulators by pairing to their target mRNAs. It is worth noting that our team led the way in finding let-7 as a critical regulator in BCSC fates—the reduced expression of let-7 could not only enhance the self-renewal of BCSCs by upregulating HRAS but also facilitate their differentiation through high levels of HMGA2; while let-7 overexpression repressed mammosphere formation, neoplasia and metastasis in NOD/SCID mice.^72^ Additional stemness-related miRNAs that we found include miR-30 and miR-34c.^73,74^ Among the miR-200 family, miR-200c targeting the self-renewal gene *Bmi-1* not only strongly prevents murine MaSCs from generating normal mammary outgrowth, but also represses tumorigenicity of human BCSCs *in vivo.*^75^ Polyl isomerase Pin1 was identified as another key target of miR-200c to regulate stemness of mouse MaSCs and human primary BCSCs,^76^ and to induce EMT, a stem cell property demonstrated in both normal and cancer stem cells.^69,77^ Also, miR-22 overexpression facilitates EMT, invasiveness and metastasis of MaSCs and BCSCs by, on the one hand, targeting TET1, TET2 and TET3, on the other hand, upregulating genes associated with stemness and EMT (for example, BMI1, ZEB1 and ZEB2).^78^ Moreover, miR-93 can modulate the fates of normal and malignant MaSCs by regulating their proliferation and differentiation states.^79^ And miR-27b is involved in the generation of BCSCs when its downregulation activates ENPP1.^80^ Our previous review has listed the reported BCSC-associated miRNAs and their functions.^81^ Here, a brief summary of miRNAs regulating the biology of BCSCs is given in Table 1.

Besides miRNAs, long noncoding RNAs (lncRNAs) emerge as new players in stem cell signaling via multiple biological mechanisms, functioning as molecular guides/decoys/scaffolds or competitive RNAs (ceRNAs) to miRNAs.^82^ Defined roles of lncRNAs in stemness signaling and lineage commitment can be exemplified by Pinky and lncTCF7 demonstrated in neural and liver cancer stem cells, respectively. The neural-specific lncRNA Pinky associates with the splicing regulator PTBP1, regulating the expression of key transcripts involved in neuronal differentiation and neurogenesis from neural stem cells.^83^ lncTCF7 promotes the self-renewal of human hepatocellular carcinoma stem cells through TCF7-activated Wnt pathway.^84^ The direct evidence of lncRNAs regulating BCSCs lies on lncRNA-ROR, whose upregulation accounts for the expansion of CD24^−^CD44^+^ cell population and the induction of EMT.^85^ Mechanically, it functions as a ceRNA to miR-205, thus preventing its target gene ZEB2, also an EMT inducer, from being degraded. Another lncRNA PINC was reported to modulate differentiation of mammary epithelial progenitors via interaction with polycomb repressive complex 2,^86^ whereas proofs of stem cell involvement are still lacking. Basically, there are various lncRNAs involved in EMT of BrCa cells, including HOTAIR, MALAT1, BCAR4 and lncRNA-ATB. However, it needs to be demonstrated with further studies that these lncRNAs can directly regulate MaSCs or BCSCs during mammary gland or BrCa development.

The technical problems such as difficulty in the precise purification of stem cells due to a lack of unique cell-surface markers may, to a large extent, restrict the identification of potent regulatory ncRNAs. But, linking stemness and EMT to specific ncRNAs will help elucidate the mechanisms of breast tumorigenesis and development, paving the path for putative therapeutic ncRNA targets to be rendered onto clinic application.

## Concluding remarks and future translational notes

In recent years, tremendous progress has been made in delineating the mammary epithelial hierarchy where mammary stem/progenitor cells drive mammary gland development and induce breast tumorigenesis upon malignant transformation. However, there are still many challenges, especially experimental techniques, lying ahead for the MaSC field. First, lacking unique cell-surface marker precludes precise purification and enrichment of MaSCs and descendent progenitors. Second, the stemness of MaSCs is hard to maintain during *in vitro* cell culture due to their predisposition to differentiation, even if using mammosphere assays. Third, lineage tracing can only be conducted in mice in the presence of established cell-lineage specific promoters for cells of interest since differences between human and rodent organs and cells cannot be ignored. Fourth, oncogenic transformation of MaSCs via lentiviral transduction is not yet achieved and requires further technical improvement. Another complex issue is the heterogeneity of MaSCs, whether, at least in part, attributed to the dedifferentiation of progenitors or more differentiated cells. Moreover, mounting evidence has suggested that the mammary microenvironment and MaSC niche may impact on mammary gland development and breast oncogenesis, for example, it still remains elusive how stromal fibroblasts or extracellular matrix contribute to normally developed and/or cancerous mammary gland. Finally, the cell hierarchy within the BrCa tissue is hitherto well-veiled: do BCSCs hold the predominance and give birth to other BrCa cells? where are BCSCs from? All these issues open wide for exploration and await more definite clarification.

Since all efforts of these translational findings aim at clinical application, it is evident that identification of the cell of origin harbors clinical implications including new preventive and/or therapeutic approaches for the onset/relapse/progression of BrCa. The novel biomarkers expressed by the cell of origin may enable earlier detection of BrCa and further effective prevention, such as chemoprevention applied in BRCA1/2 mutation carriers with high susceptibility to BrCa. Also, the altered expression of critical regulatory molecules, either proteins or ncRNAs, associated with the stemness or tumorigenicity can be clinically useful in early-diagnostic and prognostic evaluation of BrCa. Last but not the least, the gene signature of the cell of origin will help unveil key signaling pathways and initiating mutations where new targeting therapies could be built for the treatment of early-stage BrCa.

## Figures and Tables

**Table 1:** Functional miRNAs involved in BCSC biology

*Stem cell biology*	*Functions*	*miRNAs involved*
Self-renewal	Anti-BCSC	Let-7 family (let-7a, let-7b, let-7c, let-7d, let-7e, let-7f, let-7g, let-7i, miR-98), miR-200 family (miR-200a, miR-200b, miR-200c, miR-141, miR-429), miR-30 family(miR30a, miR-30b, miR-30c, miR-30d, miR-30e), miR-34c, miR34a, miR-128, miR-27b, miR-100, miR-205
	Pro-BCSC	MiR-495
Proliferation and differentiation	Anti-BCSC	Let-7 family, miR-200 family, miR-30 family, miR-34c, miR-93, miR-128, miR-16, miR-140, miR-27a, miR-205
	Pro-BCSC	MiR-93, miR-495, miR-181 family (miR-181a, miR-181b, miR-181c), miR-29, miR-142, miR-221, miR-135b
Tumorigenicity	Anti-BCSC	Let-7 family, miR-200c, miR-205
	Pro-BCSC	MiR-142
EMT	Anti-BCSC	Let-7 family, miR-200 family, miR-30 family, miR-34c, miR-7, miR-128, miR-93, miR-205
	Pro-BCSC	MiR-181, miR-21, miR-495, miR-22, miR-221, miR-9
Invasion and metastasis	Anti-BCSC	Let-7 family, miR-200a, miR-34c, miR-93, miR-7
	Pro-BCSC	MiR-22, miR-21, miR-9
Chemoresistance	Anti-BCSC	MiR-128, miR-16, miR34a
	Pro-BCSC	MiR-125b

Abbreviations: BCSC, breast cancer stem cell; EMT, epithelial to mesenchymal transition; miR, microRNA.
